# Depression, Anxiety and Stress among Nepali Health Care Workers during the Coronavirus Disease 2019 Pandemic: A Cross-sectional Survey

**DOI:** 10.31729/jnma.6747

**Published:** 2021-06-30

**Authors:** Ishwor Sharma, Anurag Misra, Bipin Kumar Shrestha, Arun Kumar Koirala, Anita Banjade, Prakash Banjade

**Affiliations:** 1 Division of Nephrology, University at Buffalo, New York, United States of America; 2 Black Country Healthcare NHS Foundation Trust, West Midlands, United Kingdom; 3 Department of Physiology, Nepalese Army Institute of Health Sciences, Kathmandu, Nepal; 4 School of Health and Allied Sciences, Pokhara University, Nepal; 5 Patan Academy of Health Sciences, Oxford University Clinical Research Unit, Nepal; 6 Emergency Medical Services, Ministry of Health, Male, Maldives

**Keywords:** *anxiety*, *COVID-19*, *depression*, *mental health*, *stress*

## Abstract

**Introduction:**

Studies among health care workers from different part of world during the coronavirus disease 19 pandemic have reported substantial impact on their physical, mental and emotional wellbeing. This study measured the impact of coronavirus disease 2019 on the mental health of Nepali healthcare workers in different parts of the world during the pandemic.

**Methods:**

This cross-sectional survey was carried out from December 25, 2020 to Jan 25, 2021. Ethical approval was taken from the Institutional Review Committee (reference number: 372). Online questionnaire including demographic profiles and Depression, Anxiety, and Stress Scales-21 instrument were sent to Nepali healthcare workers around the world through social media apps using convenience sampling. Data were entered into Microsoft Excel for Mac version 16.49 and analysed.

**Results:**

Among 208 who participated in the study, 62 (30%) participants were positive for anxiety, 47 (22.5%) for depression and 25 (12%) for stress. Higher prevalence of depression 18 (30%) and stress 10 (17%) was found in nurses compared to paramedics, among whom depression was seen in 5 (20%) and stress in 4 (16%). Among doctors, depression was found in 24 (19%) and stress in 11 (9%).

**Conclusions:**

This study demonstrated that a high proportion of healthcare workers were suffering from depression, anxiety and stress. Our findings are similar to the data from other national and international studies.

## INTRODUCTION

Coronavirus disease 2019 (COVID-19) pandemic has placed enormous strain on physical and mental health of Healthcare Workers (HCWs). Increased workhour, the risk of infection to self and family members, limited availability of Personal Protective Equipment (PPE), death of fellow health-workers etc. are the stressors affecting the HCWs.

Studies done during the pandemic of severe acute respiratory syndrome (SARS) and Ebola have reported significantly high incidence of psychological distress among healthcare workers leading to emergence of PTSD, anxiety, depression and burnout.^1^ A study conducted during SARS pandemic demonstrated 18-57% of physician with emotional distress.^2^ A study from China during COVID-19 pandemic showed 39.1% of HCWs had psychological distress, especially among those working in Wuhan.^3^

The aim of this study was to find out the prevalence of depression, anxiety, and stress among Nepali healthcare workers in different parts of the world.

## METHODS

This cross-sectional survey was conducted from Dec 25, 2020 to Jan 25, 2021. Ethical approval was taken from the Institutional Review Committee of the Nepalese Army Institute of Health Sciences (Reference no. 372). The study was done among HCWs including doctors, nurses and paramedics (health assistants, community medical assistants, lab technicians and pharmacist). The inclusion criteria consisted of all Nepali healthcare workers, ≥18 years old who gave informed consent. The exclusion criteria were those who were unable to use electronic device. Convenience sampling was done and the sample size calculated as,

$$\begin{array}{ll} \text{n} & \text{= }{\text{Z}}^{\text{2}}\times \text{p}\times \text{q}/{\text{e}}^{\text{2}}\\ & \text{= }{\left(1.96\right)}^{\text{2}}\times (0.5)\times (0.5)/{(\text{0.07)}}^{\text{2}}\\ & \text{= }194 \end{array}$$

Where,

n = minimum required sample size
Z = 1.96 at 95% Confidence Interval
p = prevalence taken as 50% for maximum sample size
q = 1-p
e = margin of error, 7%

The calculated sample size was 194 but 208 responses were included in the study. An online questionnaire was sent to health care workers currently working in Nepal and also to those originally from Nepal but working in different countries of the world. Google form was used to create the online questionnaire and messaging apps like Viber, Facebook messenger, WhatsApp and also social media like Facebook were used to disseminate the questionnaire. Demographic characteristics, medical history, validated Depression, Anxiety and Stress scales (DASS-21)^4^ instrument were included in the questionnaire.

The collected data were entered and analyzed using Microsoft Excel Sheet. The prevalence of Depression, Anxiety and Stress were calculated and binary data were expressed as frequency and percentage.

## RESULTS

**Figure 1:**
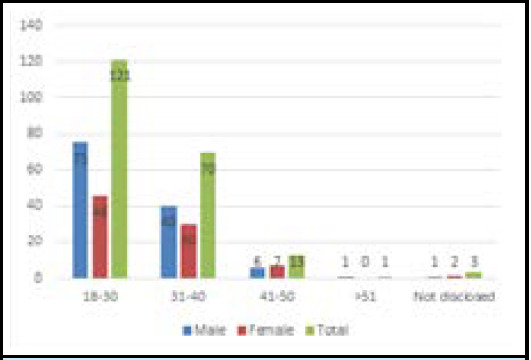
Distribution of the respondents according to age and sex.

Out of 208 HCWs who participated in the study, 94 (45.2%) have a private practice, 55 (26.7%) have their practice in government hospitals, 50 (23.8%) in academic settings and 9 (4.3%) in other nongovernment institutes. Our study showed that 170 (81.9%) of HCWs that participated in the study have Urban practice while 38 (18.1%) have Rural practice. The participants were between 18 and 51 years (Figure 1, Table 1).

**Table 1:** Participants' baseline characteristics.

Characteristics	Total (n= 208) n (%)	Doctors (n= 123) n (%)	Nurses (n=60) n (%)	Paramedics (n=25) n (%)
Gender				
Female	98 (47.1)	28 (22.7)	60 (100)	10 (40.0)
Male	110 (52.8)	95 (77.2)	0 (0)	15 (60.0)
Ethnicity				
Janajati	46 (22.1)	16 (13.0)	21 (35.0)	9 (36.0)
Khas	130 (62.5)	90 (73.1)	28 (46.6)	12 (48.0)
Madhesi	25 (12.0)	14 (11.4)	7 (11.6)	4 (16.0)
Others	7 (3.3)	3 (2.4)	4 (6.6)	0 (0)
Marital status				
Single	105 (50.4)	56 (45.5)	34 (56.6)	15 (60.0)
Married	96 (46.1)	63 (51.2)	24 (40.0)	9 (36.0)
Divorced, Separated	4(1.9)	2 (1.6)	1 (1.6)	1 (4.0)
Others	3 (1.4)	2 (1.6)	1 (1.6)	0 (0)
Practicing Country				
Nepal	167 (80.2)	106 (86.1)	44 (73.3)	17 (68.0)
United States	8 (3.8)	2 (1.6)	4 (6.6)	2 (8.0)
United Kingdom	2 (0.9)	0 (0)	1 (1.6)	1 (4.0)
Maldives	16 (7.6)	9 (7.3)	5 (8.3)	2 (8.0)
India	3 (1.4)	2 (1.6)	1 (1.6)	0 (0)
Australia	6 (2.8)	2 (1.6)	3 (5.0)	1 (4.0)
Pakistan	1 (0.4)	1 (0.8)	0 (0)	0 (0)
China	1 (0.4)	1 (0.8)	0 (0)	0 (0)
Blank	4(1.9)	0 (0)	2 (3.3)	2 (8.0)

Sixty-two (30%) participants screened positive for anxiety, 47 (22.5%) for depression and 25 (12%) for stress. The prevalence of anxiety, depression and stress was higher among female Nepalese compared to males; 41 (42%), 30 (31%), and 17 (17%) respectively (Table 2).

**Table 2:** Prevalence of depression, anxiety and stress using DASS-21^*^ among male and female Nepalese healthcare workers.

Variables	Male Healthcare personnel (n=110) n (%)	Female Healthcare personnel (n=98) n (%)	Total n (%)
Depression	17 (15.5)	30 (30.6)	47 (22.5)
Anxiety	21 (19.0)	41 (42.0)	62 (30)
Stress	8 (7.0)	17 (17.3)	25 (12)

* DASS-21 = Depression, Anxiety, and Stress Scales.

**Figure 2:**
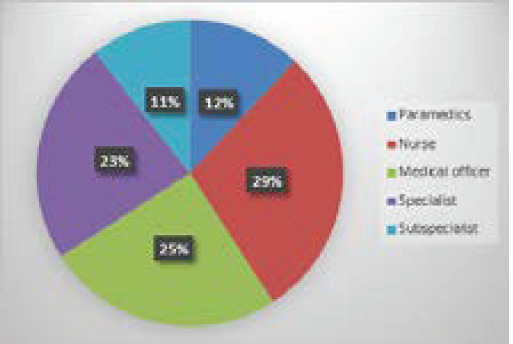
Profession of the respondents.

Among the three groups of healthcare workers, 28 (47%) nurses, seven (28%) paramedics and 27 (22%) doctors screened positive for anxiety (Figure 2).

Depression was noted to be higher 18 (30%) in nurses followed by paramedics in 5 (20%) and Doctors 24 (19%). Stress was also higher among the Nurses 10 (17%) compared to paramedics 4 (16%) and doctors 11 (9%) (Table 3).

**Table 3:** Prevalence of Depression, Anxiety and Stress using DASS-21 among Nepalese Healthcare workers.

Prevalence n(%)	Classification DASS-21	Doctors (n=123)	Nurses (n=60)	Paramedics (n=25)
Depression	Normal	99 (80.4)	42 (70)	20 (80)
	Mild	14 (11)	10 (17)	2 (8)
	Moderate	7 (6)	5 (8)	3 (12)
	Severe	1 (0.8)	1 (1.6)	0 (0)
	Extremely severe	2 (1.6)	2 (3.2)	0 (0)
Anxiety	Normal	96 (78)	32 (53)	18 (72)
	Mild	17 (14)	15 (25)	0 (0)
	Moderate	4 (3)	5 (8)	4 (16)
	Severe	2 (1.6)	3 (5)	0 (0)
	Extremely severe	4 (3)	5 (8)	3 (12)
Stress	Normal	112 (91)	50 (83)	21 (84)
	Mild	5 (4)	7 (12)	2 (8)
	Moderate	3 (2)	1 (1.6)	2 (8)
	Severe	2 (1.6)	1 (1.6)	0 (0)
	Extremely severe	1 (0.8)	1 (1.6)	0 (0)

The stress was related to lack of personal protective equipment (PPE) in 159 (76.44%) participants administrative load in 118 (56.73%) participants, taking care of critical patients in 113 (54.32%) participants and lack of hospital resources in 140 (67.3%) participants. The financial stressors in HCWs were also noted. Pay cut and decreased income due to COVID-19 was noted in 102 (49%), job loss in 62 (30%) and stock market loss in 96 (46.15%). The majority 107 (51.2%) of HCW's who responded were single. Married respondents were 95 (45.9%) and rests 6 (2.9%) were divorced, separated or widowed.

## DISCUSSION

Our study showed participation of 208 Healthcare Workers among 300 of those who were invited, roughly equating to 69%, which signifies adequate participation when compared to other studies. Similar study done in the Middle East showed a response rate of 71.9%, which also signifies that, our study participation represented a significant response rate.^5^ Our study showed a significantly greater male HCWs participation. Out of 208 respondents, 110 (52.8%) were male and 98 (47.1%) were female.

A study in 2003 in Toronto, during SARS outbreak, showed that two-thirds of the teaching hospital staff surveyed had increased levels of concerns for personal and family health, and almost one-third of a subset of respondents were emotionally distressed.^6^ Based on assessment by DASS-21 used in our study, 134 (64.5 %) of the participants suffered either from depression 47 (22.5%), anxiety 63 (30%) or stress related symptoms 24 (12%). This demonstrates that our study result is in accordance with the similar study done in the past in Toronto.

In a meta-analysis conducted with studies from December 2019 to May 2020, most studies reported a higher prevalence of anxiety (30% to 70%) and depressive symptoms (20% to 40%). In those studies, insomnia, burnout, emotional exhaustion or somatic symptoms were also reported.^7^ Similar to this, our study demonstrated that 63 (30%) participants screened positive for anxiety, 47 (22.5%) for depression and 24 (12%) for stress.

In a study conducted in China (Hubei province), depression (46.9%), anxiety (41.1%), insomnia (32%), and stress (69.1%) were demonstrated among registered nurses and physicians. Moreover, frontline health care providers had clinically significant levels of depression, anxiety, insomnia, and stress compared to the non-frontline health care providers.^8^ Our study had a similar association but lower prevalence of depression, anxiety and stress compared to the Hubei, which was the epicenter of the disease. This may be because of the lesser number of cases and less severe COVID-19 cases taken care by the study respondents in our study.

Another survey of 1257 nurses and physicians caring for patients with COVID-19 in China found that healthcare providers (41.5% of respondents) had significantly more depression, anxiety, insomnia and distress than providers who did not care directly for the patients.^9^ Similarly, an observational study in China of 180 health care workers providing direct care for patients with COVID-19 found considerable levels of anxiety and stress that profoundly affected their sleep quality and self-efficacy.^10^ Our study did not compare the difference in the emotional well-being among frontline health care workers versus those who were not directly involved in the care of COVID-19 patients. However, 64.5% of the respondents have reported minor to major level of emotional distress as aforementioned, which is noteworthy.

In this study, 159 (76.44 %) reported significant stress secondary to lack of PPE and 140 (67.3 %) reported stress secondary to inadequate hospital resources. These results reflect the need to provide adequate safety and precautionary measures to lessen the probability of the emotional stress among HCWs and prioritize adequate resources including professional support for the well-being of HCWs during pandemic. The pandemic has far reaching effects in the life of HCW's. The psychological well-being is interconnected to the economic effects like pay cut and job loss. Likewise, undue pressure from hospital administration to work without appropriate PPE's and also the severity of the disease they deal with may have acute and chronic mental effects.

Our study had certain limitations. It did not compare difference in emotional stress among frontline healthcare workers who were directly involved in the care of COVID-19 patients to those who were not. The study also did not look into the long-term effects on the mental health of HCW's like post-traumatic stress disorder. Similarly, our study did not evaluate the difference of emotional stress among HCWs of different ethnic groups, urban and rural practice and those practicing in Nepal and abroad.

## CONCLUSIONS

Our study demonstrated that a high proportion of healthcare workers were in psychological distress during the COVID-19 outbreak. Proper and effective intervention at a personal and institutional level to mitigate the adverse psychological and emotional effect on HCWs due to COVID-19 is essential to support them from stress, anxiety, depression and burnout. Healthcare workers are the pillars of health delivery of every country; thus, their emotional wellbeing is crucial in delivering adequate health care during the time of pandemic. The early recognition and intervention of psychological stress among HCWs along with the prevention of such stress by adequately addressing perceived factors including availability of PPE and adequate hospital resources are the key factors for successfully handling the pandemic while mitigating the adverse mental effects of the health care workers.
